# Artificial and Bioengineered Therapeutic Options for Corneal Endothelial Disease

**DOI:** 10.3390/bioengineering12101064

**Published:** 2025-09-30

**Authors:** Lanxing Fu, Alfonso Vasquez Perez, Sundas Maqsood, Nick Kopsachilis, Roberta Foti, Fabiana D’Esposito, Mutali Musa, Daniele Tognetto, Caterina Gagliano, Marco Zeppieri

**Affiliations:** 1 Department of Ophthalmology, East Kent Hospitals University NHS Foundation Trust, Canterbury CT1 3NG, UK; l.fu@nhs.net (L.F.);; 2 Cornea and External Disease Department, Moorfields Eye Hospital NHS Foundation Trust, London EC1V 2PD, UK; 3 Department of Ophthalmology, Maidstone and Tunbridge Wells NHS Foundation Trust, Maidstone ME16 9QQ, UK; 4 Division of Rheumatology, A.O.U. “Policlinico-San Marco”, 95123 Catania, Italy; 5 Department of Medicine and Surgery, University of Enna “Kore”, 94100 Enna, Italy; 6 Imperial College Ophthalmic Research Group (ICORG) Unit, Imperial College, London NW1 5QH, UK; 7 Department of Optometry, University of Benin, Benin 300283, Nigeria; 8 Department of Ophthalmology, Centre for Sight Africa, Nkpor, Onitsha 434112, Nigeria; 9 Department of Medicine, Surgery and Health Sciences, University of Trieste, 34129 Trieste, Italy; 10 Eye Center, “G.B. Morgagni-DSV”, 95125 Catania, Italy; 11 Department of Ophthalmology, University Hospital of Udine, 33100 Udine, Italy

**Keywords:** artificial cornea, endothelial keratoplasty, tissue engineering, biomimetic scaffolds, corneal endothelial dysfunction

## Abstract

*Background*: Corneal endothelial dysfunction continues to be a primary indication for corneal transplantation globally. Due to ongoing constraints in donor tissue availability and graft durability, artificial graft technologies are increasingly recognized as viable alternatives, particularly for eyes unsuitable for conventional allogeneic transplantation. *Aim*: This article examines the contemporary state of artificial corneal endothelial grafts, emphasizing technological advancements, incorporation into surgical procedures, and their developing function in meeting the unfulfilled requirements of endothelial keratoplasty. *Methods*: A comprehensive synthesis of recent preclinical and clinical literature was performed, concentrating on scaffold-based constructs, cell-seeded and acellular methodologies, biomaterial characteristics, and innovative surgical delivery techniques. The review highlights translational pathways and contrasts the initial outcomes of artificial and donor-derived endothelial grafts. *Results*: Advancements in regenerative biomaterials and cell culture systems have resulted in the development of functional endothelial substitutes. Engineered grafts, comprising decellularized stromal carriers, synthetic polymer matrices, and human cell-laden constructs, have demonstrated promising biocompatibility and functional results in preliminary trials. The integration of these constructs into methods akin to Descemet membrane endothelial keratoplasty (DMEK) has improved clinical viability, diminished immunologic risk, and shown potential for visual recovery. *Conclusions*: Artificial endothelial grafts signify a revolutionary advancement in corneal surgery, addressing donor shortages and expanding the applications of endothelial keratoplasty. Although additional clinical validation and regulatory processes are required, existing evidence indicates that these technologies may soon transform treatment protocols for corneal endothelial disease.

## 1. Introduction

Corneal endothelial dysfunction, encompassing disorders such as Fuchs’ endothelial corneal dystrophy (FECD) and pseudophakic bullous keratopathy, constitutes a significant cause of visual impairment and blindness globally [1,2]. Corneal disease has been described as the fifth leading cause of global blindness, affecting more than 10 million people [2]. The corneal endothelium is a non-proliferative monolayer of hexagonal cells that preserves corneal transparency via an essential pump-and-barrier mechanism. The corneal endothelium does not regenerate, unlike the epithelium. When the endothelial layer fails, stromal edema develops, resulting in a loss of corneal clarity and severe visual deterioration. For decades, the definitive treatment has been corneal transplantation, with full-thickness penetrating keratoplasty (PK) being the standard technique since the first successful case in 1905 [3]. Over the past two decades, partial-thickness lamellar transplantation techniques have been introduced that are less invasive and have led to more rapid visual recovery.

Endothelial keratoplasty, such as Descemet stripping automated endothelial keratoplasty (DSAEK) and Descemet membrane endothelial keratoplasty (DMEK), is now the mainstay of surgical treatment for primary corneal endothelial dysfunction [4]. These two techniques differ by the amount of donor tissue grafted; DMEK is an anatomical replacement of the Descemet membrane and endothelium, whereas DSAEK includes an additional layer of posterior donor stroma. DSAEK was rapidly adopted following the introduction of the technique by Melles due to the visual and safety benefits it offers in comparison with PK [5,6]. DMEK was first described in 2006 by Melles and was adopted due to its better visual outcomes compared to DSAEK and a lower rate of immunological rejection [7]. However, DMEK surgery is technically more challenging with a learning curve associated with higher rates of primary graft failure and rebubbling [8,9]. The number of DMEK grafts performed has steadily increased as more surgeons have been trained and the availability of pre-peeled and pre-loaded DMEK tissue has become more widespread [10].

There is a greater risk of graft failure in more complex eyes, such as those requiring repeat grafting. The limitations of PK in these eyes have encouraged alternatives to be developed, such as the Boston type 1 keratoprosthesis (Boston KPro, Massachusetts Eye and Ear Infirmary, Boston, Massachusetts). Over 20,000 KPro devices have been implanted since their introduction in the 1960s [11]. Visual rehabilitation is complex even with a clear graft, with topical antibiotic medication and bandage contact lens use to maintain the ocular surface and management of secondary glaucoma. Current commercially available devices demonstrate significant complications with longer follow-up durations [12,13,14]; hence, artificial corneas are often utilized as a last resort option for end-stage corneal disease, following multiple failed conventional grafts.

These keratoplasty procedures still rely on the availability of healthy human donor corneas, a resource that is critically limited globally. The worldwide demand for donor tissue significantly exceeds the supply, with half of the world lacking access to donor corneal tissue [15]. This shortage is exacerbated in developing countries that have a higher burden of corneal blindness but without the infrastructure to support a corneal transplantation programme. There are only a few countries that can export donor corneas significantly (US, Sri Lanka, Italy), with others not able to meet their domestic demand [16]. Conditions that result in limbal stem cell deficiency, such as chemical injuries and autoimmune diseases, may not be suitable for PK or the Boston KPro, which still require carrier donor tissue.

In response to this significant clinical challenge, there has been ongoing interest in developing fully synthetic devices, with or without the addition of corneal endothelial cells, to enhance graft availability and reduce the complication profile. These novel methods utilize advancements in tissue engineering, biomaterials, and cell culture techniques to provide functional alternatives to donor tissue. These grafts encompass bioengineered constructions, wherein human corneal endothelial cells are grown and then deposited onto ultra-thin biocompatible scaffolds, as well as acellular matrices and synthetic polymer films intended to facilitate host cell integration or serve as functional replacements. The primary objective is to provide a conveniently accessible, standardized, and immunologically compatible alternative that can be included in contemporary minimally invasive surgical procedures.

This review aims to provide a comprehensive overview of these cutting-edge technologies, synthesizing the findings from pivotal preclinical and clinical studies to evaluate their design, surgical applications, and transformative potential in redefining the standard of care for corneal endothelial disease.

## 2. Literature Search Strategy

This study was designed as a narrative review to summarize and critically evaluate the existing evidence on bioengineered methods for corneal endothelium restoration. A thorough literature search was conducted utilizing PubMed, MEDLINE, Scopus, and Web of Science to discover pertinent preclinical and clinical studies in the field. The search method utilized combinations of terms such as artificial cornea, corneal endothelial dysfunction, endothelial keratoplasty, tissue engineering, corneal endothelial cells, biomimetic scaffolds, and decellularized corneal matrix. Where applicable, Medical Subject Headings (MeSH) and Boolean operators were utilized to expand the breadth and enhance retrieval sensitivity. The search was unrestricted by publication year to encompass both the evolution of previous experimental techniques and the latest translational research, with the last update occurring in July 2025. To maintain evaluative consistency, only papers published in English were included.

The article selection method adhered to the criteria of transparency and reproducibility as advocated for narrative reviews. All collected titles and abstracts were meticulously examined, and studies considered pertinent were obtained in their entirety. The selection concentrated on articles detailing the design, manufacture, and evaluation of artificial corneal endothelium grafts, including both cellular and acellular structures, biomaterial scaffolds, and polymeric alternatives. Special emphasis was placed on research documenting translational viability, including in vitro characterization of endothelial cell functionality, ex vivo assessments in human or animal corneas, and in vivo evaluations in preclinical or clinical environments. Reports restricted to conjectural comments or solely theoretical modeling were excluded.

The literature examination was performed separately by two reviewers, with discrepancies in interpretation reconciled through consensus. No explicit risk of bias instrument was utilized; nonetheless, methodological attributes including sample size, repeatability, control conditions, and translational potential were included in the evidence synthesis. The merits and weaknesses of each study were evaluated alongside their experimental outcomes. This investigation aimed to deliver an integrated and descriptive perspective instead of a pooled quantitative analysis; thus, the data were synthesized thematically. Evidence was categorized into domains that mirrored the natural evolution of the field, commencing with the advancement of culture techniques for corneal endothelial cells, succeeded by delivery strategies, the fabrication of scaffolds and biomaterials, and ultimately the preliminary clinical applications of artificial endothelial grafts. In each topic, the discourse on results highlighted both scientific advancement and the methodological limitations that may affect the interpretation of findings. This methodology facilitated a critical and thorough account of the translation of experimental research into early clinical practice, simultaneously highlighting the existing gaps that must be addressed for general acceptance.

## 3. Techniques for Sourcing and Culturing Corneal Endothelial Cells

The first protocol for human corneal endothelial cell (hCEC) expansion in vitro was reported in 1965 [17]. Several steps are necessary for culture and expansion: donor tissue selection, removal of the donor endothelium and DM, isolation of the hCECs by enzymatic or non-enzymatic digestion, seeding the cell suspension with culture media and growth factors, and expansion on substrates mimicking the in vivo environment [18]. Primary CECs are commonly derived from cadaveric donor corneas, and stem cells (adipose tissue, umbilical cord blood, bone marrow), embryonic stem cells, and hCEC precursors [19,20,21,22,23]. DM with endothelium is peeled from the cadaveric donor, and separation of the hCECs from the DM is achieved by tissue digestion. Enzymatic digestion requires collagenase, dispase, and trypsin, whereas ethylenediaminetetraacetic acid (EDTA) is used in non-enzymatic digestion to dissociate cell junctions [24,25]. The cellular morphology of the CECs must be maintained during culture, as intracellular pathways can be activated, leading to the CECs transitioning to a fibroblast-like phenotype (epithelial–mesenchymal transition; EMT) and a loss of function [26,27,28]. Numerous culture media options have been tested for the expansion of CECs. Dual media utilizes one medium with abundant growth factors to encourage CEC proliferation and another medium supplemented with serum, but without growth factors, to avoid EMT [29,30]. Optimized culture media typically contain growth factors such as basic fibroblast growth factor (bFGF), epidermal growth factor (EGF), transforming growth factor-beta (TGF-β), fetal bovine or human serum albumin, extracellular matrix (ECM) components, antibiotics, antimycotics, osmolarity, and pH controls.

The addition of Rho-associated protein kinase (ROCK) inhibitors affects cell migration, adhesion, and cytoskeleton organization, enhancing cell–cell adhesion and promoting proliferation [31]. Improving the confluency and polarity of CECs by increasing the seeding density during culture also reduces the risk of EMT [26]. Advances in culture techniques include perfusion-based systems that ensure a continuous supply of nutrients and concurrent waste disposal, as well as three-dimensional (3D) systems that are more similar to the in vivo CEC environment [32]. The CECs can be grown on tissue culture plates, but reports have suggested that coating the plates yields better results [25,33,34]. Common coating choices include collagen, albumin, and fibronectin, which are often admixed with collagen [33,34,35]. These proteins interact with integrin receptors on CECs, promoting cell adhesion and survival [36,37,38,39,40]. Mechanical stimulation, such as substrate stiffness and fluid shear stress, can promote more uniform CEC alignment and proliferation, thereby mimicking the natural corneal tissue architecture necessary for transparency [41,42,43,44,45].

A primary obstacle to the production of clinical-grade corneal endothelial cells (CECs) has been the propensity of cultured cells to undergo endothelial-to-mesenchymal transition (EndMT; referred to as epithelial–mesenchymal transition, EMT, in the CEC literature), resulting in the adoption of fibroblast-like morphology, cytoskeletal reorganization, and the loss of junctional integrity and pump function markers [26]. EndMT/EMT has been linked to inadequate biochemical and biophysical signals in vitro, characterized by excessive mitogen exposure, TGF-β signaling, low seeding density, and non-physiological substrate stiffness. These factors result in polygonal loss, polymegathism/pleomorphism, decreased ZO-1 continuity, and reduced Na^+^/K^+^-ATPase expression [26]. Simultaneously, age-dependent replicative constraints and stress-induced senescence have restricted expansion potential; cells from younger donors have enhanced proliferative capacity, while those from older donors demonstrate diminished cycling and a tendency toward senescent phenotypes [28]. EndMT/EMT and senescence are critical failure modes that must be managed in any manufacturing practice (GMP) aligned process designed for clinical transplantation.

A dual-media strategy has been employed to diminish EndMT/EMT while attaining scale, wherein CECs are alternated between a proliferation-permissive medium (abundant in growth factors) and a stabilization medium (serum-supplemented but reduced or devoid of growth factors) to restore a quiescent, barrier-competent phenotype prior to harvest [29]. This cycle has facilitated expansion during the development phase and subsequently mitigated EndMT/EMT danger during stabilization, resulting in enhanced maintenance of hexagonal shape and junctional protein expression [29]. In addition to media cycling, GSK3 inhibition has become a method to promote expansion while preserving chromosomal stability and endothelial identity; pharmacological GSK3 blockade has augmented the proliferation of human corneal endothelial cells (CECs) and produced cultures with a normal karyotype and functional characteristics indicative of pump-barrier competence [46]. The integration of media-stage modulation (dual-media) and pathway-targeted small-molecule control (GSK3 inhibition) provides a logical framework to alleviate EndMT/EMT and senescence issues, thereby enhancing the viability of generating standardized, transplant-grade CEC sheets or suspensions [29,47].

It is crucial to recognize that numerous studies detailing endothelial cell expansion are constrained by restricted sample quantities and short-term culture validation. External validation of protocols among independent laboratories remains limited, and reporting standards exhibit significant variability. Common risks include spectrum bias, poor management of class imbalance in cell morphology, and insufficient information regarding missing data, with just a minority of studies offering calibration or repeatability measures. These constraints indicate that although early culture techniques seem promising, their applicability to standard clinical environments should be approached with caution.

## 4. Methods of Administration for Corneal Endothelial Cell Therapy

Cell injection therapy and cell sheet transplantation are two methods for delivering CEC to the recipient cornea. Injection therapy consists of injecting CECs that have been cultured, sometimes with a ROCK inhibitor, directly into the anterior chamber without a carrier scaffold. The first human study describing this technique was conducted by Kinoshita et al. in 11 patients with bullous keratopathy [48]. Prone positioning was required for several hours after injection to encourage CEC adhesion to the posterior cornea [49]. Normal corneal endothelial function and clarity were restored in 10 out of 11 eyes with a mean endothelial cell density of 1257 cells/mm^2^ (601–2067 cells/mm^2^) at 5-year follow-up [48]. Cadaveric donor corneas unsuitable for conventional keratoplasty can be used for in vitro CEC expansion. Parikumar et al. reported visual improvement in three bullous keratopathy patients when using CEC cultured from discarded donor corneas with nanocomposite gel sheets to enhance cell adhesion [50].

These initial small-scale studies have shown promising results; however, safety remains a consideration. Given that the injected CECs are not secured to a scaffold, the fate of unattached CECs is unknown. There is a potential risk that the unattached CECs can reach the trabecular meshwork and the systemic circulation, causing tumour formation [51]. To mitigate these risks, research has been undertaken to develop functional scaffolds that imitate the ECM of the native DM to encourage cellular regeneration [52]. These engineered scaffolds serve as templates, providing adhesion, migration, and proliferation support for cells. DM is formed of collagen IV-VIII, laminin, and fibronectin, as well as other ECM components, and supports CEC growth and function [32,53]. Alterations of the DM are a cause of common endothelial diseases such as FECD [18]. Therefore, replicating the DM’s properties is desirable to optimize the delivery of CECs [53,54].

Notwithstanding promising first clinical findings, methodological shortcomings are apparent. Numerous studies have involved minimal patient cohorts lacking control groups, and long-term follow-up is constrained. In numerous instances, safety evaluations regarding the disposition of non-adherent cells or the potential for intraocular problems have not been methodically quantified. There is a deficiency in external validation of results across several sites, and spectrum bias may occur due to the selective selection of patients with reasonably intact ocular anatomy. These restrictions limit the robustness of conclusions regarding the dependability and scalability of delivery strategies at this juncture.

## 5. Composition and Fabrication of Scaffolds for Endothelial Replacement

Different types of materials have been utilized for scaffold engineering in corneal regeneration therapy. These include natural materials such as chitosan, hyaluronic acid, alginate, cellulose, collagen, gelatin, and silk [52,53,54,55]. Polycaprolactone (PCL), polyvinyl alcohol (PVA), and polyethylene glycol (PEG) are some of the synthetic biopolymers available. It is crucial to match cell needs with the optimal scaffold materials, as cells can exhibit differences in function among different materials [56]. Hydrophilic materials (collagen, gelatin, chitosan, and PEG) will encourage attachment to the posterior corneal surface, which is essential for graft survival [57]. It is common to combine natural and synthetic materials, thereby overcoming the limitations of individual materials.

Chitosan and collagen can mimic the ECM and reduce inflammation and infection risk, whereas PCL and PEG are excellent for mechanical strength. Chitosan-PCL material combines chitosan nanoparticles that improve cellular attachment with the addition of PCL, which can enhance chitosan’s molecular properties [56]. The transparency of the composite chitosan-PCL material is related to lower PCL content. Rabbit CECs have demonstrated good attachment and proliferation on a composite material made of gelatin, chondroitin sulfate, and hydroxyethyl chitosan [58,59]. Incorporating chitosan improves the elasticity and durability of the composite material, and further studies can focus on decreasing the membrane thickness. The feasibility of CEC transplantation using these artificial membranes has been demonstrated using sheep eyes. Ocelli et al. created a 50 μm thick chitosan-PEG hydrogel film that had high permeability, optical transmission, and allowed cultured sheep CECs to be transplanted in ex vivo trials [60].

It has been shown that transplanted decellularized donor DM after descemetorhexis encourages CEC migration by acting as a natural scaffold [61,62]. The porcine cornea has a similar function and biology to the human cornea and exists in greater abundance [63]. Porcine stroma and decellularized DM have been used for keratoplasty in several studies [63,64,65]. Biomaterials used to fabricate scaffold membranes include human and animal corneal DM and placental amniotic membrane [55,66]. DM properties, such as biomechanics, need to be preserved during the decellularization process [67,68,69,70]. The decellularized membranes require testing to assess their tensile strength (resistance to rupture and deformation) and evaluate light transmission and transparency with spectrophotometric analysis. Decellularized membranes will have reduced immunogenicity, thereby encouraging better biocompatibility and facilitating surgical manipulation during keratoplasty. The limitations of this option include reliance on donor cornea supply, variability in donor membrane compositions, risks of contamination and infection, and loss of growth factors, cytokines, and proteins during the decellularization process, which are important for corneal health [69,71]. Establishing a standardized decellularization protocol for human corneas that removes all cellular materials and maintains membrane integrity is necessary for this scaffold fabrication technique to become more mainstream [71].

Three-dimensional (3D) fabrication has been explored with bioprinting to create custom products using layer-by-layer generation from computer-aided design (CAD) models. Most studies have utilized 3D bioprinting to replicate corneal epithelium and stroma. The anatomical and mechanical features of DM, such as its thickness, are challenging to replicate using current 3D bioprinting technology. Material customization is necessary to replicate human tissue characteristics that are suitable for different 3D bioprinting techniques [72,73]. The first attempt at 3D bioprinting hCECs utilized extrusion-based techniques to deposit hCECs within a gelatin-based bioink onto a decellularized amniotic membrane in an animal model [74]. The hCECs were transfected with ribonuclease 5 to enhance their survival capacity and increase proliferation [74]. Grönroos et al. bioprinted CECs derived from human pluripotent stem cells contained in a covalently crosslinked hyaluronic acid bioink [75]. The structures expressed phenotypic markers, including Na^+^/K^+^ ATPase [75]. To date, there have been no clinical trials with 3D printed endothelial constructs. Synthetic scaffold-like implants are already commercially available and have shown early efficacy in the treatment of corneal endothelial dysfunction.

The variety of scaffold materials and manufacturing techniques demonstrates the inventive potential of the sector, yet presents obstacles for standardization and comparison. Research frequently employs varying objectives for biocompatibility and mechanical evaluation, complicating cross-study comparisons. A multitude of research remain in the preclinical stage, with inconsistent reporting of light transmission, tensile strength, and immunogenicity. Moreover, reproducibility is constrained by limited sample sizes and variability in decellularization methods, which lack standard validation. Consequently, whereas scaffolds exhibit significant conceptual and experimental promise, their clinical relevance necessitates careful interpretation until more consistent approaches are developed. An overview of notable preclinical and clinical studies on cell-based and scaffold-supported endothelial therapies is included in Table 1.

The examined studies collectively demonstrate significant advancements in bioengineering techniques for corneal endothelium replacement, although they also exhibit persistent methodological shortcomings that must be acknowledged when evaluating the findings. The majority of investigations are limited in scope and often lack external validation, resulting in uncertainty regarding reproducibility among laboratories and clinical settings. The reporting of critical characteristics, including patient selection, follow-up duration, and management of incomplete data, is frequently variable, and standardized outcome measures are rarely utilized. Furthermore, the prevalence of case reports, pilot studies, and short-term experimental models indicates that long-term safety and durability have not been fully explored. These limitations highlight the necessity for careful interpretation of the existing data and stress the importance of standardizing study design and reporting to facilitate more dependable comparisons and future meta-analyses.

## 6. Artificial Therapeutic Options for Corneal Endothelial Disease

### Surgical Feasibility, Graft Survival, Postoperative Complications

The EndoArt (EyeYon Medical, Ness Ziona, Israel) exemplifies an advanced prototype of bioengineered endothelial substitutes, although it must be considered within a broader framework of advancements, including cell-based therapies, scaffold-assisted transplantation, and hybrid approaches.

**Figure 1:**
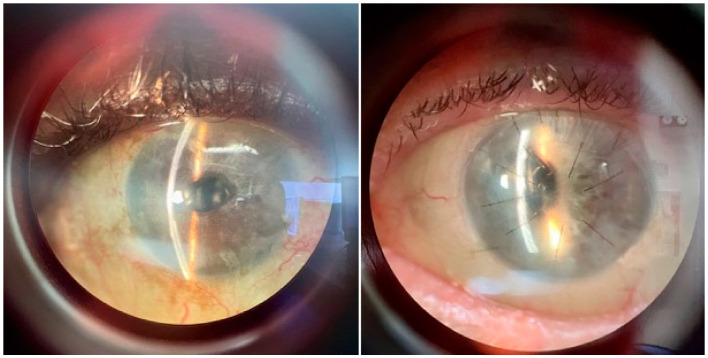
The EndoArt was implanted in an eye with a glaucoma drainage device (**left**) and an anterior chamber lens with anchoring sutures in situ (**right**).

EndoArt is a 50 μm 6.5 mm disc made from a copolymer of hydroxyethyl methacrylate and methyl methacrylate, has been developed to treat corneal endothelial dysfunction. It has recently received breakthrough designation by the FDA (Food and Drug Administration) in the US. The mechanism of action is as a barrier to excess fluid hydration in the posterior stroma in eyes with endothelial failure. Diseases causing chronic corneal edema, such as pseudophakic bullous keratopathy or previously failed conventional grafts, are suitable candidates. The EndoArt is suitable for eyes with a history of repeated failed grafts, uveitis, glaucoma drainage devices, or those with unstable anterior chambers (Figure 1), as it has been shown that conventional grafts in these eyes exhibit reduced graft survival over time [76,77,78]. The surgical technique is similar to that of endothelial keratoplasty, with a Descemetorhexis performed to remove the host Descemet’s membrane and endothelium. The EndoArt has an “F” mark to aid in correct orientation within the anterior chamber. Anchoring sutures are used to secure the EndoArt, as cases had high detachment and rebubbling rates; these are removed at 3–6 months postoperatively [79,80]. A bandage contact lens (BCL) placed at the end of surgery is used to enhance ocular surface healing.

A multicentre clinical trial involving 52 patients was recently completed, and the results will be scrutinized for any deviation from real-world cases in the literature [81]. The first case was described in 2021 [82], and a summary of published studies with outcomes is summarized in Table 1. Long-term graft survival for DSAEK and DMEK is comparable in reported studies, with the majority of cases exceeding 90% in non-complex eyes [83,84,85,86,87]. In eyes at high risk of graft rejection and failure, such as redrafts, glaucoma drainage devices, silicone tamponade, and anterior chamber intraocular lenses, the graft survival rate of conventional endothelial keratoplasty is significantly lower [77,78,88]. It remains to be seen whether the EndoArt or similar implants in the future can match the long-term graft survival of conventional endothelial keratoplasty.

There have been no reports of a lack of biocompatibility, anterior chamber inflammation, vascularization, or corneal melt [89]. There have been a few cases reported with continual corneal stromal thinning up to 17 months after surgery [89,90]. Secondary raised intraocular pressure has not been a major postoperative feature following EndoArt implantation. There was one reported case of endophthalmitis 7 months following EndoArt implantation, but a causal link was not identified [91]. Explantation was reported in 6 eyes out of 24 by Daphna et al., due to persistent detachment after rebubbling when descemetorhexis was not undertaken (*n* = 5), and due to intraocular lens dislocation and inability to form an adequate air bubble [92]. The major complication is the higher rate of graft detachment and the need for rebubbling (Table 2).

**Figure 2:**
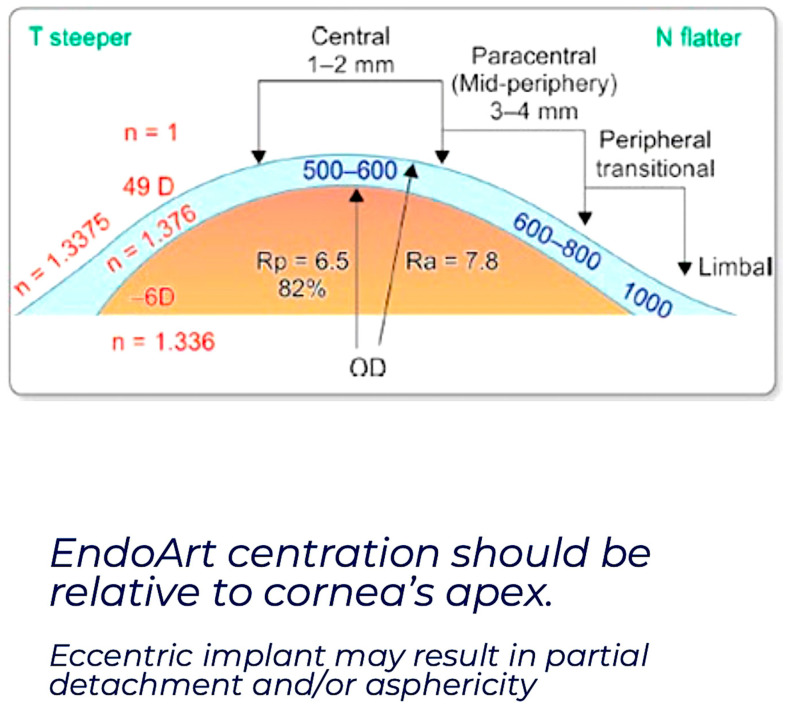
It is essential to centralise the placement of the EndoArt implant as eccentric placement can result in partial or complete detachment.

Reported rates of rebubbling range from 0–100%; however, this could be due to a combination of the surgeon learning curve, lack of centration (Figure 2) and the reduced flexibility of the device compared with DMEK tissue, and its ability to conform to the rapidly changing profiles in the posterior stroma [93]. There have been two reported cases of spontaneous reattachment without the need for rebubbling [94]. Anchoring sutures can be used to secure the EndoArt (Figure 1) and do not affect the survival of the graft, even though there are no endothelial cells attached to the device. Further studies examining the use of and optimal number and configuration of anchoring sutures, along with their removal schedule, are needed to promote EndoArt adhesion.

Besides EndoArt, other novel artificial constructs for endothelial replacement have recently been created. Fang et al. (2024) described the fabrication of a transparent amphiphilic artificial corneal endothelium layer made from hydroxyethyl methacrylate and ethylene glycol dimethylacrylate, featuring a hydrophilic surface coated with polyvinylpyrrolidone [95]. In a rabbit model of bullous keratopathy, this flexible and biocompatible membrane exhibited robust adherence to the posterior corneal surface during gas tamponade, maintenance of normal corneal architecture, and low inflammation for a period of 100 days. This method emphasizes the promise of non-cellular, donor-independent endothelial substitutes as feasible alternatives to traditional grafts, expanding the range of artificial therapeutic possibilities being explored.

While EndoArt has demonstrated promising preliminary outcomes, its existing evidence base is equivalent in scale and maturity to that of other modalities, such as cell injection therapy and scaffold-supported endothelial replacement. Table 1 presents a comparative analysis that highlights the necessity for direct trials and extended follow-up to ascertain the relative advantages of each technique. Clinical data for artificial endothelium implants, such as the EndoArt, primarily originate from case reports or short series. The durations of follow-up are inconsistent, and the incidence of postoperative complications, especially detachment and rebubbling, varies significantly across research. A multitude of published studoes exhibit a deficiency in standardized outcome reporting, complicating the comparison of visual acuity and corneal thickness among cohorts. Spectrum bias may exist, as these implants are frequently tested on highly selected patients with intricate ocular histories, which may not represent the wider candidate population. Collectively, these characteristics indicate that the observed success of surgical feasibility and graft survival should be regarded as preliminary evidence of feasibility rather than conclusive confirmation of long-term clinical dependability.

**Table 2:** Summary of published studies reporting EndoArt outcomes.

Study	Study Type	Number of Eyes	Pre-op CCT (µm)	Percentage Reduction of CCT	Visual Acuity Improved	Final Visual Acuity (LogMAR)	Indication for Surgery	Co-Morbidities	Postop Complications
Auffarth et al., 2021 [82]	Case report	2	745 ± 22	34%	n/a	n/a	Failed DMEK	Case 1: treated endophthalmitis	Rebubbling rate 100%
Abusayf et al., 2023 [96]	Case report	1	911	24%	Yes	0.7	PBK	Glaucoma, previous vitrectomy	No
Kobayashi et al., 2024 [79]	Case report	1	845	37%	Yes	2	Failed DMEK	Epiretinal membrane	Rebubbling
Wiedemann et al., 2024 [97]	Case series	3	719 ± 145	18%	Yes	1.1 ± 0.6	GDD, failed DMEK	Glaucoma	Raised IOP (*n* = 1) CMO (*n* = 1) Subepithelial corneal opacity (*n* = 1) Rebubbling rate 33%
Romano et al., 2024 [80]	Case report	2	887 ± 268	30%	Yes	Case 1: 0.30 Case 2: 1.70	Failed DSAEK and PK		No
Fontana et al., 2025 [98]	Case series	7	805 ± 131	28%	Yes	0.95 ± 0.28	Failed DMEK, DSEK, GDD	Glaucoma	Rebubbling rate 57%
Daphna et al., 2025 [92]	Clinical Trial	24	759 ± 116	19%	Yes	1.34 ± 0.57	Failed DMEK, DSEK, DSO	Glaucoma, pars plana vitrectomy, macular disease	Explantation rate 25% Rebubble rate 2.9 ± 2.0 procedures per patient

Abbreviations: PBK, pseudophakic bullous keratopathy; GDD, glaucoma drainage device; PK, penetrating keratoplasty; DSAEK, Descemet stripping automated endothelial keratoplasty; DMEK, Descemet membrane endothelial keratoplasty; DSO, Descemet stripping only; LogMAR, logarithm of the minimum angle of resolution; IOP, intraocular pressure; CMO, cystoid macular oedema; CCT, central corneal thickness; n/a, not applicable or data not provided.

## 7. Limitations of Current Evidence and Future Directions

### 7.1. Methodological Limitations of Current Evidence

The advancement of bioengineered endothelial grafts signifies a notable scientific progress; nonetheless, the existing evidence remains constrained in both scope and rigor. Numerous studies depend on limited cohorts, frequently comprising fewer than twelve patients or experimental eyes, so constraining the robustness of the results that can be derived. Single-center studies predominate in the literature, and results have seldom been replicated in independent environments. Furthermore, the majority of papers include only short- to mid-term follow-up, so hindering an evaluation of long-term safety and durability. In the absence of standardized comparison arms or matched control groups, it is challenging to differentiate genuine therapeutic effects from enhancements that may arise from case selection or surgical proficiency.

The absence of standardized reporting protocols among studies is equally significant. Crucial methodological specifics, including patient selection criteria, graft preparation processes, and outcome definitions, are frequently inadequately detailed, hence constraining the reproducibility of results. Factors such as absent data, outcome calibration, and adverse event reporting are inconsistently managed, and this variability hinders the synthesis of evidence across many publications. Collectively, these constraints emphasize the necessity for prudent interpretation of the existing literature and reinforce the significance of establishing standardization for forthcoming research in this domain.

### 7.2. Regulatory and Translational Considerations

The use of artificial endothelial grafts in normal practice will rely on stringent regulatory control and the standardization of manufacturing protocols, alongside scientific and surgical advancements. Currently, there is no globally recognized protocol for the preclinical validation of bioengineered corneal structures. Research utilizes diverse biomaterials, culture conditions, and implantation techniques; nevertheless, safety evaluations are not conducted based on uniform criteria. This diversity presents issues for regulatory organizations responsible for assessing new devices and may postpone clinical implementation. Consistently validating biocompatibility, mechanical integrity, and optical performance will be essential for establishing the credibility of these technologies.

A crucial factor is the scalability of production and distribution. Laboratory approaches that exhibit success on a small scale may not be easily applicable to large-scale production under good manufacturing practice (GMP) standards. Variations in scaffold composition, cellular origins, and surgical delivery techniques must be considered within a regulatory framework that guarantees reproducibility, safety, and quality assurance. Establishing explicit translational paths that encompass preclinical testing, early-phase clinical trials, and regulatory approval processes will be imperative. Only through this collaborative endeavor will artificial endothelium grafts advance from promising experimental structures to safe and effective therapeutic alternatives in routine ophthalmic treatment.

### 7.3. Future Research Directions

Future research in this domain must tackle the methodological and translational deficiencies that presently hinder broad implementation. Extensive, multicenter clinical trials with prolonged follow-up durations are necessary to yield dependable data on transplant longevity, endothelial cell density, and long-term safety. Comparative studies that directly juxtapose bioengineered structures with traditional donor-derived grafts are essential, since they provide objective result benchmarking and assess whether artificial substitutes may genuinely equal or exceed the performance of human tissue. Standardization of study design, with explicit definitions of objectives and consistent reporting of problems, will be crucial for facilitating meaningful comparisons across various investigations.

Concurrent initiatives should concentrate on enhancing the fundamental biology and material science principles of these constructions. Improvements in scaffold composition, particularly through the creation of biomimetic materials that more accurately emulate the natural Descemet’s membrane, may enhance cell adherence and functionality. Improvements in culture methodologies, like the incorporation of perfusion systems or three-dimensional culture conditions, may augment the stability of enlarged corneal endothelial cells. Mitigating variability in decellularization and bioprinting methods is essential for guaranteeing reproducibility. Through the amalgamation of stringent clinical trials and ongoing advancements in biomaterials and cell biology, next research will bridge the divide between experimental potential and clinical applicability, facilitating the dependable incorporation of bioengineered endothelium grafts into corneal surgery.

This narrative review is limited by the constraints of its non-systematic approach. Notwithstanding the comprehensive examination of many databases and the incorporation of the most relevant studies, there remains a likelihood that some publications were neglected. The variety of study designs, ranging from preclinical laboratory research to tiny pilot clinical series, hindered direct outcome comparisons and the use of stringent risk of bias assessments. Furthermore, much of the available information consists of case reports or limited studies with short follow-up durations, which restricts the ability to draw solid conclusions about long-term safety and efficacy. These limitations must be recognized when evaluating the findings presented in this review.

## 8. Conclusions

The compilation of studies demonstrates progress in producing clinically viable artificial corneal endothelial grafts. Advancements in biomaterial science have resulted in the development of ultra-thin, transparent scaffolds that effectively replicate the original Descemet’s membrane. Preclinical investigations consistently reveal that these biomimetic scaffolds support robust adhesion, proliferation, and maintenance of the unique polygonal morphology and high cell density of cultivated human CECs.

Cell-based techniques have shown great promise, with numerous studies reporting the successful generation of functional endothelial cell layers from both primary human coronary endothelial cells (hCECs) and cultured cell lines. The principal problem persists in the restricted proliferative ability of mature hCECs; however, methodologies utilizing Rho-associated kinase (ROCK) inhibitors have markedly enhanced cell expansion in vitro. Early-phase clinical trials testing these bioengineered grafts have indicated favorable outcomes. In specific patient groups exhibiting endothelial dysfunction, the implantation of these constructions resulted in notable corneal transparency, an enhancement in visual acuity, and stable endothelial cell counts over the first follow-up intervals.

The clinical translation of these grafts has been substantially simplified by their compatibility with existing minimally invasive surgical procedures. Most artificial grafts are designed for distribution via small self-sealing wounds utilizing injectors similar to those employed in DMEK. This method minimizes surgical trauma, reduces the likelihood of postoperative problems, including excessive astigmatism, and accelerates visual recovery. The integration with current surgical workflows not only enhances the safety profile but also reduces the learning curve for surgeons, thereby driving wider clinical adoption.

## Figures and Tables

**Table 1:** Summary of representative cell-based and scaffold-supported therapies for corneal endothelial disease (excluding EndoArt).

Modality	Study (Year)	Design	Model/ Population	N (Eyes)	Scaffold/ Carrier	Key Procedural Details	Follow-Up	Main Efficacy Outcomes	Safety/Adverse Events	Key Limitations
Cell injection therapy (hCEC + ROCK inhibitor)	Kinoshita et al. (2018) [48]	Prospective, single-arm clinical study	Human, bullous keratopathy	11	None (cell suspension)	Intracameral injection of cultured hCECs with ROCK inhibitor; prolonged prone positioning	5 years	Restoration of clarity in 10/11 eyes; mean ECD ≈ 1257 cells/mm^2^ (range 601–2067)	No major safety signal reported by authors	Small cohort; no control; specialized postop positioning; generalizability uncertain.
Cell injection—prone time optimization	Mimura et al. (2007) [49]	Preclinical	Rabbit model	—	None	Evaluated necessary prone time after cell delivery	Acute	Identified posture-dependent adhesion dynamics	Preclinical only	Translational parameters extrapolated to humans.
Cell sheet on nanocomposite gel	Parikumar et al. (2024) [50]	Clinical case series	Human, post-PK bullous keratopathy	3	Nanocomposite gel sheet	Transplantation of cultured hCECs expanded from discarded corneas on gel sheets	16-year follow-up reported	Sustained corneal stability and visual improvement reported	Long duration single-center; device/material specifics unique	Very small sample; selection bias; lacks standardized endpoints.
Allogeneic Descemet’s membrane scaffold to promote host CEC monolayer	Bhogal et al. (2017) [61]	Experimental (ex vivo/in vivo translational)	Post-descemetorhexis models	—	Donor Descemet’s membrane	DM transplantation as “soil” to guide endothelial monolayer restoration	Short- to mid-term	Enhanced monolayer formation and functional restoration signals	Immunologic and variability concerns inherent to biologic scaffold	Non-standardized models; clinical evidence pending.
Decellularized stroma/DM (porcine or human) as ultrathin substrate for endothelial sheets	Du & Wu (2011) [55]; Zhang et al. (2022) [63]	Preclinical	Porcine/human tissues; animal implantation	—	Decellularized full-thickness porcine matrix; ultrathin acellular porcine stroma	Scaffold fabrication, optical/mechanical testing; seeding with hCEC	Short-term	High transparency; good permeability; supported CEC adhesion/proliferation	Potential residual immunogenicity if decellularization incomplete	Heterogeneous protocols; no clinical trials yet.
Synthetic/biohybrid thin films (e.g., chitosan-PEG; chitosan/PCL; gelatin-based composites)	Ozcelik et al. (2013) [58]; Tayebi et al. (2021) [52]	Preclinical	Ex vivo / animal	—	Chitosan-PEG hydrogel; chitosan/PCL; gelatin–chondroitin sulfate–hydroxyethyl chitosan	Fabrication of ultrathin transparent films; CEC seeding; ex vivo transplant feasibility	Short-term	High optical transmission; adequate permeability; CEC attachment and proliferation demonstrated	Material-specific risks; mechanical handling vs. DM; regulatory path unknown	Mostly bench/ex vivo; limited in vivo duration.
Bioprinted endothelial constructs (hPSC-derived or hCEC-based)	Kim et al. (2018) [74]; Grönroos et al. (2024) [75]	Preclinical	Animal/ex vivo; in vitro	—	Decellularized amniotic membrane or HA-based bioink	Extrusion bioprinting of endothelial layers; RNASE5 overexpression to enhance survival; hydrazone-crosslinked HA for hPSC-CEC bioprinting	Short-term	Expression of endothelial markers (e.g., Na^+^/K^+^-ATPase); viability and sheet formation	No clinical translation yet; bioink standardization pending	Early-stage feasibility; durability and function in vivo not established.
Induced pluripotent stem cell–derived CECs	Ng et al. (2023) [41]	Preclinical review and experimental reports	In vitro	—	Various	Differentiation protocols for iPSC-to-CEC; delivery concepts	—	Phenotypic and functional characteristics reported	Genetic/epigenetic stability; scalability	Heterogeneous methods; clinical trials lacking.
ECM-mimetic substrates/coatings to stabilize phenotype and reduce EMT	Peh et al. (2015) [29]; Koo et al. (2014) [37]	Preclinical	In vitro hCEC culture	—	Collagen, fibronectin, poly-ε-lysine hydrogels; micro/nanotopography	Dual-media propagation; ECM coatings; topography-guided culture	—	Improved proliferation with preserved morphology; reduced EMT risk	Culture-to-clinic translation untested	Surrogate outcomes; lacks clinical endpoints.

Abbreviations: hCEC, human corneal endothelial cell; ECD, endothelial cell density; DM, Descemet’s membrane; EMT, endothelial-to-mesenchymal transition (epithelial–mesenchymal transition usage varies in literature); PK, penetrating keratoplasty; ROCK, Rho-associated protein kinase; HA, hyaluronic acid.

## Data Availability

No new data were created or analyzed in this study. Data sharing is not applicable to this article.
